# The mitochondrial genome and phylogenetic position of a conehead katydid *Euconocephalus pallidus* (Insecta: Orthoptera)

**DOI:** 10.1080/23802359.2022.2054373

**Published:** 2022-03-25

**Authors:** Renke Dong, Jingyou Chen, Fengxin Liu, Shen Zhu, Shuhong Hou, Wen Lin, Sheng Li, Na Li

**Affiliations:** a School of Life Sciences, Guangdong Provincial Key Laboratory of Insect Developmental Biology and Applied Technology, Institute of Insect Science and Technology, South China Normal University, Guangzhou, China; b Guangmeiyuan R&D Center, Guangdong Provincial Key Laboratory of Insect Developmental Biology and Applied Technology, South China Normal University, Meizhou, China

**Keywords:** Mitochondrial genome, conehead katydid, *Euconocephalus pallidus*

## Abstract

The complete mitochondrial genome of a conehead katydid *Euconocephalus pallidus* was determined. The mitochondrial genome is 15,888 bp in size with an A + T content of 71.67%. It contains 13 protein-coding genes, 22 transfer RNA genes, two ribosomal RNA genes, and a control region. The order and orientation of these genes conform to the ancestral form of insects. Phylogenetic analysis supports a close relationship between *E*. *pallidus* and *E*. *nasutus*.

*Euconocephalus pallidus* (Redtenbacher, 1891), belonging to the subfamily Conocephalinae (Insecta: Orthoptera), is a conehead katydid with considerable economic importance. The species is distributed mainly in tropical and subtropical areas, including China, India, Pakistan, and Singapore (Shi et al.^ 2001^; Shishodia et al. 2010; Tan 2011). *E*. *pallidus* and its congeneric species, e.g. *E*. *nasutus*, are quite similar in morphology. To provide genetic data that could contribute to species identification and phylogenetic analysis, we report the complete mitochondrial genome (mitogenome) of *E*. *pallidus*.

Fresh samples of *E*. *pallidus* were collected from Wuzhi Mountain, Hainan Island, China (18.777° N, 109.517° E) and preserved in 100% ethanol at 4 °C. Whole genomic DNA was extracted from a hind leg of a single specimen using a DNeasy Blood & Tissue kit (Qiagen, Hilden, Germany). The specimen and DNA (voucher number EP10) were deposited in Guangdong Provincial Key Laboratory of Insect Developmental Biology and Applied Technology, Institute of Insect Science and Technology (Na Li, lina5hs@m.scnu.edu.cn). The specimen handling and experimental procedures were in compliance with the Guide to Animal Experiments of the Ministry of Science and Technology (Beijing, China). The genomic DNA was subjected to high-throughput sequencing on the HiSeq 2500 platform (Illumina Inc., San Diego, CA). The full mitogenome was assembled with MITObim v1.9 (Hahn et al. 2013), annotated on the MITOS webserver (Bernt et al. 2013), and deposited in GenBank (accession number MW009066). To validate the control region sequence, PCR amplification followed by the Sanger sequencing was performed.

The complete mitogenome of *E*. *pallidus* is a circular molecule of 15,888 bp in length with an A + T-biased nucleotide composition (36.85% A, 34.83% T, 17.58% C, and 10.75% G). The mitogenome is compact with 11 intergenic spacers totaling 73 bp and nine overlaps totaling 32 bp between adjacent genes. It has a non-coding control region, which is 1029 bp in length and contains 2.0 tandem copies of a 260-bp sequence, and encoded a typical set of 37 genes, i.e. 13 protein-coding genes, 22 transfer RNA genes, and two ribosomal RNA genes (Table S1). The gene composition, order, and orientation are identical to the ancestral forms of insects. The 22 transfer RNA genes range in length from 63 bp (*trnA*) to 72 bp (*trnV*). Canonical ATN codons serve as start codons of 11 protein-coding genes whereas *cox1* and *nad1* start with CCG and GTG, respectively. Six protein-coding genes terminate with a complete stop codon TAA, while *cob*, *cox1*, *cox2*, *cox3*, *nad4*, *nad5*, and *nad6* end with incomplete stop codons (T or TA). Comparison of the 13 protein-coding gene sequences reveals a total of 380 synonymous and 40 nonsynonymous substitutions between *E*. *pallidus* and *E*. *nasutus* (NC053383). These variations result in a genetic distance ranging from 0.027 for *nad3* to 0.060 for *cob*.

Phylogenetic relationships of the subfamily Conocephalinae were reconstructed in MrBayes v3.2 (Ronquist et al. 2012) and IQ-TREE (Minh et al. 2020) based on concatenated data of the 13 protein-coding genes and two ribosomal RNA genes. The genes were each aligned using MUSCLE (Edgar 2004), filtered by Gblocks (Castresana 2000), and concatenated using Perl scripts (Minxiao et al. 2011). Best-fit partitioning strategies and molecular evolution models for these genes were determined in PartitionFinder v2.1.1 (Lanfear et al. 2017). The Bayesian inference and the maximum likelihood method produced identical tree topologies (Figure 1). Both trees support the monophyly of the subfamily and the monophyly of its two tribes (Conocephalini and Copiphorini). The newly sequenced *E*. *pallidus* is sister to *E*. *nasutus* with a high posterior probability of 1.00 in the Bayesian inference tree and a bootstrap value of 100% in the maximum-likelihood tree. In conclusion, the mitogenome sequence obtained in our study represents essential data for species identification of *E*. *pallidus* and phylogenetic analysis of Conocephalinae.

**Figure 1.**
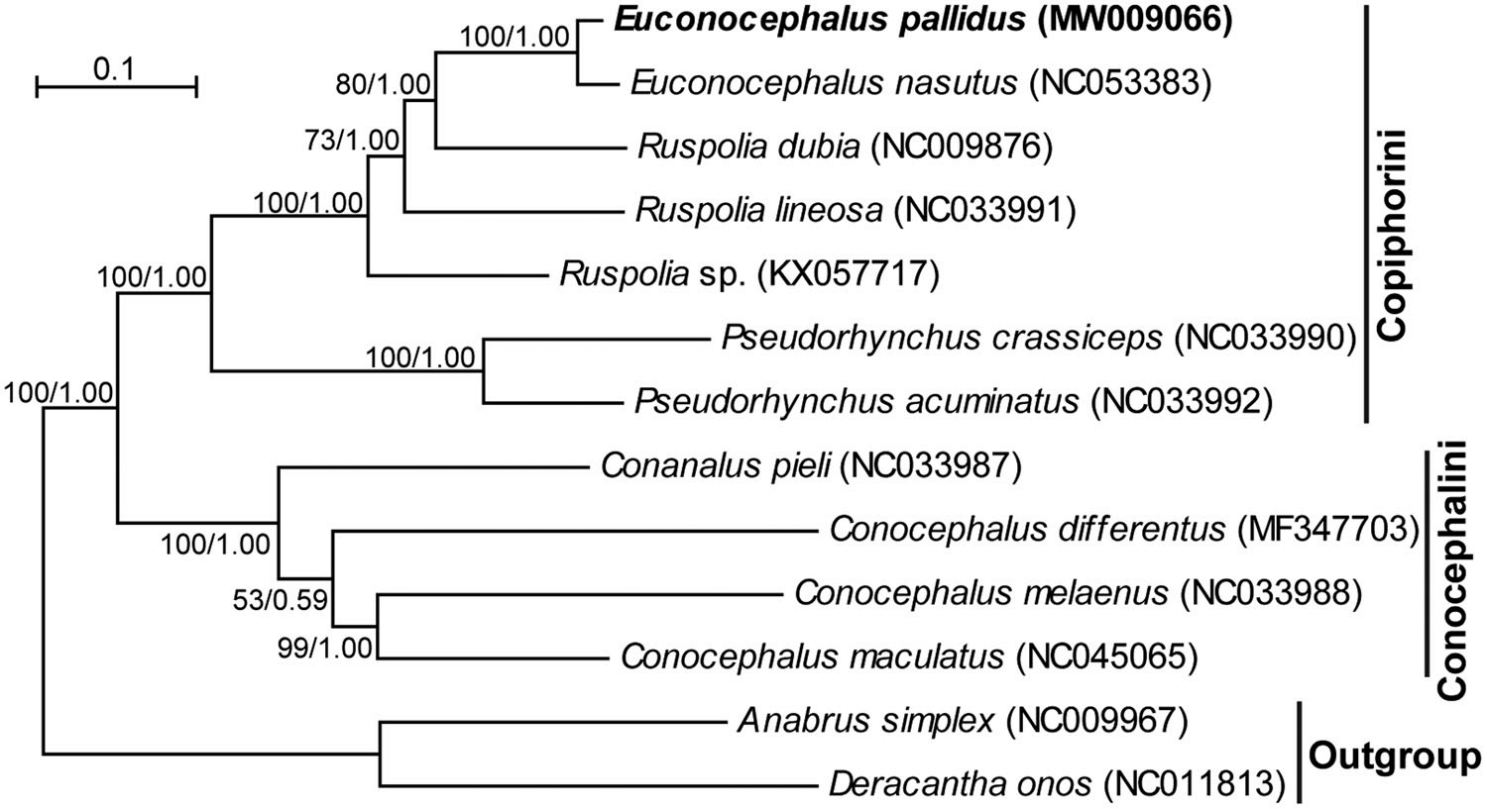
The maximum-likelihood tree of the subfamily Conocephalinae based on the 13 protein-coding genes and two ribosomal RNA genes. *Anabrus simplex* and *Deracantha onos* from closely related subfamilies served as outgroup taxa to root the tree. Bootstrap values (in percentage) for the maximum-likelihood method and Bayesian’s posterior probability values for the Bayesian inference are shown at nodes. GenBank accession numbers for each taxon are provided in parenthesis.

## Authors contributions

NL conceived the study. RD, JC, and FL performed the experiment and analysis. SZ, SH, WL, and SL contributed to analysis and interpretation of the data. RD drafted the manuscript. All authors revised and approved the final manuscript.

## Supplementary Material

Supplemental Material

## Data Availability

The data that support the findings of this study are openly available in GenBank of NCBI at https://www.ncbi.nlm.nih.gov, reference number MW009066. The associated BioProject, SRA, and BioSample numbers are PRJNA756612, SRR15616352, and SAMN20893093, respectively.
